# Early development of treatment motivation predicts adherence and symptom reduction in an internet-based guided self-help program for binge eating disorder

**DOI:** 10.3389/fpsyt.2022.969338

**Published:** 2022-10-06

**Authors:** Eik Runge, Esben Kjems Jensen, Kim Mathiasen, Pia Veldt Larsen, Søren Peter Thygesen Hertz, Trine Theresa Holmberg, Kristine Tarp, Jakob Linnet, Mia Beck Lichtenstein

**Affiliations:** ^1^Mental Health Services in the Region of Southern Denmark, Research Unit for Digital Psychiatry, Centre for Digital Psychiatry, Odense, Denmark; ^2^Department of Clinical Research, Faculty of Health Sciences, University of Southern Denmark, Odense, Denmark; ^3^Mental Health Services in the Region of Southern Denmark, Vejle, Denmark; ^4^Clinic on Gambling- and Binge Eating Disorder, Department of Occupational and Environmental Medicine, Odense University Hospital, Odense, Denmark

**Keywords:** iCBT, motivation, adherence – compliance – persistence, binge eating disorder (BED), early measurements

## Abstract

**Objective:**

Lack of motivation is widely acknowledged as a significant factor in treatment discontinuity and poor treatment outcomes in eating disorders. Treatment adherence is lower in internet-based treatment. The current study aimed to assess the relationship between treatment motivation and treatment outcomes in an internet-based therapist-guided intervention for Binge Eating Disorder (BED).

**Method:**

Adults (*N* = 153) with mild to moderate symptoms of BED participated in a 10-session internet-based treatment program. Baseline and between-session scores of “Readiness to change” and “Belief in change” were used to predict treatment completion and eating disorder symptom reduction (EDE-Q Global, BED-Q, and weekly number of binge eating episodes) at post-treatment.

**Results:**

Baseline treatment motivation could not predict treatment completion or symptom reduction. Early measures of treatment motivation (regression slope from sessions 1–5) significantly predicted both treatment completion and post-treatment symptom reduction. “Belief in change” was the strongest predictor for completing treatment (OR = 2.18, 95%-CI: 1.06, 4.46) and reducing symptoms (EDE-Q Global: *B* = −0.53, *p* = 0.001; number of weekly binge eating episodes: *B* = 0.81, *p* < 0.01).

**Discussion:**

The results indicated that patients entering online treatment for BED feel highly motivated. However, baseline treatment motivation could not significantly predict treatment completion, which contradicts previous research. The significant predictive ability of early measures of treatment motivation supports the clinical relevance of monitoring the development of early changes to tailor and optimize individual patient care. Further research is needed to examine treatment motivation in regard to internet-based treatment for BED with more validated measures.

## Introduction

Binge eating disorder (BED) is the most common specific eating disorder (1, 2), with a global lifetime prevalence of 2.8% for women and 1.0% for men (3). BED is characterized by recurring episodes of binge eating accompanied by loss of control and followed by shame and discomfort. Binge eating episodes are usually characterized by eating rapidly, eating until uncomfortably full, and eating alone due to embarrassment. Binge eating episodes are not associated with compensatory behaviors in BED, unlike Bulimia Nervosa (4).

Binge eating disorder can be treated effectively with cognitive behavioral therapy (CBT) (5). Studies investigating internet-based CBT (iCBT) for BED have also shown promising results (6, 7). iCBT is an efficient way for broad dissemination of evidence-based treatments (8, 9) that might help break through barriers to seeking treatment (10), e.g., psychological barriers such as stigma and shame, and practical barriers such as cost, transportation, time, and inaccessibility to treatment (11). However, iCBT programs for psychological disorders such as phobic or panic disorders, post-traumatic stress disorder, insomnia, etc., tend to suffer from high rates of non-adherence (12), including internet interventions for eating disorders with drop-out rates ranging from 9 to 47.2% (13).

Treatment motivation is an ambiguous term that may predict treatment-related behaviors, such as adherence, compliance, and treatment engagement. Regarding treatment motivation in individuals already receiving treatment, the term is defined as the motivation to engage in treatment or a specific behavioral change. Previous studies have often failed to separate motivation from behavior, where treatment motivation often has been regarded as the engagement (behavior) itself, causing conceptual confusion (14).

Literature on treatment motivation in eating disorders suggests that low degrees of baseline treatment motivation predict unfavorable treatment outcomes and high drop-out rates (15–19). However, the majority of the reviews are based on studies with pre-post designs and, thus, do not include the development of motivation during treatment. A study by Vall and Wade (20) indicated that early symptom change is a strong predictor for post-treatment and follow-up outcomes in eating disorders.

In traditional face-to-face treatment for eating disorders, a majority of studies mainly emphasize treatment motivation in relation to Anorexia Nervosa and Bulimia Nervosa, while only two studies have focused on BED (18). Further, there is a lack of research examining treatment motivation in internet-based interventions for BED. More research is needed to determine ways to predict and prevent non-completion in iCBT (21).

This study aimed to investigate the association of (1) baseline treatment motivation and (2) early changes in treatment motivation with (a) treatment adherence and (b) eating disorder symptoms at post-treatment in an internet-based treatment of mild to moderate BED.

We hypothesized that baseline treatment motivation and the development of early measures of treatment motivation could predict treatment adherence and reduction in eating disorder symptoms.

## Method

### Study design

The study was an observational cohort study, which utilized collected data from an online treatment for BED. This implies that data was not collected for the purpose of the current study and its aims, and measures in the present study were not originally designed to answer the current research aims.

### Participants and recruitment

A total of 153 patients with mild to moderate BED had started treatment when data were extracted. Of these, 100 had completed the full treatment program (completion of 8–10 sessions). Patients had self-referred for treatment through an online questionnaire. A team of psychologists evaluated the answered online questionnaire for patient inclusion, hosted on the website of the Center for Digital Psychiatry, Odense, Denmark. Inclusion criteria were access to a technology device (tablet or computer), capability to read and write in Danish, age of 18 or above, mild to moderate symptom severity of BED, and absence of severe comorbidity. Patients with sub-threshold BED (BED-Q < 10) or severe BED (BED-Q > 21) were excluded. All communication regarding screening and inclusion took place online over secure mail systems and required no face-to-face contact.

### The iBED program

iBED is a 10-session text-based iCBT program for mild to moderate BED developed in 2019 by clinical psychologists in the Center for Digital Psychiatry, Mental Health Services in the Region of Southern Denmark. The program includes psychoeducation and exercises such as establishing a problem- and goal list, stable eating pattern, emotion regulation and new coping strategies. Some sessions include diaries that the patients must complete consecutively for a week. Table 4 shows a complete description of the treatment content in chronological order.

**TABLE 1:** Participant characteristics at baseline (at time T1).

Characteristic	Total	Completers	Non-completers	*P*-value
Total, n	153	100	53	N/A
Sex, n (%)				
Female	135 (88.2)	89 (89.0)	46 (86.8)	0.687
Male	18 (11.8)	11 (11.0)	7 (13.2)	–
Age, mean (SD)	39.0 (11.2)	39.5 (11.6)	38.0 (9.6)	0.476
BMI, mean (SD)	37.8 (9.6)	37.0 (9.6)	39.3 (9.4)	0.171
MDI, mean (SD)	23.1 (8.1)	22.6 (7.9)	24.2 (8.4)	0.196
Eating disorder symptoms, mean (SD)				
EDE-Q global	3.7 (0.8)	3.7 (0.8)	3.8 (0.8)	0.381
BED-Q	17.2 (2.9)	17.0 (3.1)	17.6 (2.7)	0.193
Number of binge eating episodes during the past 7 days^a^	3.8 (2.3)	3.8 (2.3)	3.9 (2.3)	0.640
Treatment motivation, mean (SD)				
Belief in change^a^	6.7 (2.3)	7.0 (2.2)	6.2 (2.4)	0.034
Readiness for change^a^	8.6 (1.8)	8.8 (1.8)	8.4 (1.8)	0.135

^*a*^Measured at Session 1.

BMI, body mass index; MDI, major depression inventory; EDE-Q, eating disorder examination – questionnaire; SD, standard deviation; N/A, not appropriate.

**TABLE 2:** Associations between completion and belief in change and readiness for change, *n* = 153.

	Completer	Non-completer	OR per unit increase	Adjusted^a,b^	
				
	Mean (SD)	Mean (SD)		OR per unit increase (95%-CI)	*P-value*
**Belief in change**					
At Session 1	7.01 (2.16)	6.23 (2.41)	1.17	1.16 (0.99, 1.35)	0.066
Patient slope (B) estimate from Sessions 1 to 5*^c^*	0.17 (0.66)	−0.02 (0.047)	1.57	2.18 (1.06, 4.46)*	0.034*
**Readiness for change**					
At Session 1	8.75 (1.85)	8.42 (1.79)	1.10	1.09 (0.91, 1.32)	0.347
Patient slope (B) estimate from Sessions 1 to 5*^d^*	−0.09 (0.59)	−0.17 (0.42)	1.27	1.25 (0.61, 2.53)	0.543

^*a*^Adjusted for sex, age, and BMI and MDI at T1. Robust standard errors are applied in adjusted analyses.

^*b*^Analyses including slope estimates are further adjusted for the measure at Session 1.

^*c*^Slope of *Belief in change* is the increase in *Belief in change* per 10 days.

^*d*^Slope of *Readiness for change* is the increase in *Readiness for change* per 10 days.

*p < 0.05.

OR, odds ratio; SD, standard deviation; B, patient linear regression slope; BMI, body mass index; MDI, major depression inventory.

**TABLE 3:** Associations between eating disorder outcomes and *Belief in change* and *Readiness for change*.

	EDE-Q global score at T2, *n* = 85			BED-Q score at T2, *n* = 85			Weekly number of binge eating episodes at Session 10, *n* = 93		
			
	Coefficient B	Adjusted^a,b^		Coefficient B	Adjusted^c,b^		Coefficient B	Adjusted^d,b^	
		Coefficient B (95%-CI)	*P*-value		Coefficient B (95%-CI)	*P*-value		Coefficient B (95%-CI)	*P*-value
**Belief in change**									
At Session 1	−0.09	−0.05 (−0.14, 0.05)	0.340	0.02	0.09 (−0.29, 0.47)	0.644	−0.08	−0.11 (−0.32, 0.10)	0.305
Patient slope (B) estimate from Sessions 1 to 5*^e^*	−0.36*	−0.53 (−0.85, −0.22)**	0.001**	−1.07	−1.20 (−2.58, 0.17)	0.086	−0.57*	−0.81 (−1.38, −0.23)**	0.006**
**Readiness for change**									
At Session 1	0.03	0.03 (−0.10, 0.15)	0.672	0.14	0.28 (−0.23, 0.79)	0.281	−0.10	−0.10 (−0.46, 0.25)	0.569
Patient slope (B) estimate from Sessions 1 to 5*^f^*	−0.47 *	−0.46 (−0.82, −0.10)*	0.012*	−0.55	−0.41 (−1.92, 1.11)	0.596	−0.27	−0.54 (−1.27, 0.19)	0.147

^*a*^Adjusted for age, sex, and EDE-Q global score, BMI and MDI at T1.

^*b*^Analyses including slope estimates are further adjusted for the measure at Session 1.

^*c*^Adjusted for age, sex, and BED-Q score, BMI and MDI at T1.

^*d*^Adjusted for age, sex, number of binge eating episodes at Session 1, and BMI and MDI at T1.

^*e*^Slope of *Belief in change* is the increase in *Belief in change* per 10 days.

^*f*^Slope of *Readiness for change* is the increase in *Readiness for change* per 10 days.

*p < 0.05.

**p < 0.01. SD, standard deviation; B, patient linear regression slope; BMI, body mass index; MDI, major depression inventory; EDE-Q, eating disorder examination – questionnaire; BED-Q, binge eating disorder – questionnaire.

**TABLE 4:** Summary of treatment-related content and tasks in the iBED program.

Session number	Content of session
Session 1	Write pros and cons for binge eating in order to prepare for behavioral change.
Session 2	Establish a problems-and-goals list for treatment.
Session 3	Complete a food-diary for 7 consecutive days in order to gain insight and understanding of personal eating pattern.
Session 4	Complete a food diary for 7 consecutive days with focus on establishing a regular eating pattern.
Session 5	Monitor, register, and describe incidences of binge eating episodes for a week.
Session 6	Establish a list of cognitive and behavioral alternative strategies to cope with cravings to binge eat.
Session 7	Use alternative strategies (from previous session) for a week and evaluate on them.
Session 8	Establish a list of compassionate self-care strategies that can strengthen self-esteem and self-image.
Session 9	Establish a list of strategies and activities to prevent relapse.
Session 10	Evaluate treatment, including achievement of personal treatment goals, and write a farewell letter to the binge eating disorder.

**FIGURE 1:**
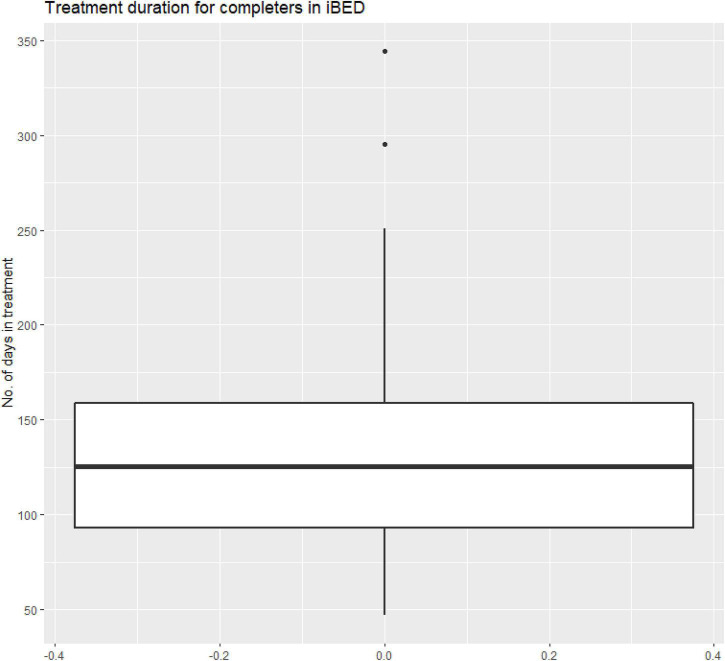
Treatment duration for completers in iBED.

Patients complete the exercises independently and receive written feedback from a psychologist. The feedback will be received no later than a week after session completion. There is no time limit for completing the sessions; however, patients will be notified when they have been inactive for more than a week. These notifications are delivered from the therapist through an asynchronous message function, which also enables the patients to deliver messages to the therapist at any time point. The messages may contain questions and comments about feedback, treatment content and exercises, binge eating episodes, motivational issues, etc. Therapists must answer the asynchronous messages no later than 5 days after they are received. Patients are required to complete the sessions sequentially before moving on to the next; however, previously completed sessions may be repeated with agreement from the therapist. Thus, the treatment completion time varied among treatment completers with a minimum to maximum range of 47 to 250 days, and with an average of 230 days (two outliers had longer durations). Figure 1 shows the distribution of treatment completion time in treatment completers.

### Assessments

Between each session, patients reply to a short questionnaire probing the following subjects during the last week: (1) number of binge eating episodes, (2) readiness to change, and (3) belief in change. All questions, except 1 number of binges, are answered on a slider ranging from 0 (not at all ready to change, no belief in change) to 10 (maximal motivation to change, maximal belief in change).

### Assessment schedule

Patients reply to the major questionnaires (BED-Q, EDE-Q, and MDI) when applying for treatment (T1) and again upon completing the full treatment program (T2). During the treatment, patients reply to the short battery of questions between each session (Symptom monitoring; SM), probing the number of binges (on a continuous scale), readiness to change, and belief in change. The two latter are single-item questions measured on a scale from 0 (not at all ready to change, no belief in change) to 10 (maximal motivation to change, maximal belief in change). Patients reply to the SM questionnaire ten times during treatment. The first response is between Session 0 and Session 1, and the last response is between Session 9 and Session 10. The time patients spend on each session varies. Consequently, the time between SM responses varies (e.g., one patient may complete session 2 in 3 days and then receive SM3, while another spends 9 days working through Session 2 before receiving SM3). The time between responses affects the slope of the linear regression. Therefore, SM responses are indexed by time. SM1 is considered day zero for a patient’s treatment, and each subsequent SM response is treated as X days after SM1 for statistical purposes. Table 5 illustrates the assessment schedule.

**TABLE 5:** Overview of assessments during treatment.

Time points	Questionnaires
T1 – Application for treatment	BED-Q, EDE-Q, MDI
Session 0	n/a
SM1	Number of binges, belief in change (0–10), readiness to change (0–10)
Session 1	n/a
SM2	Number of binges, belief in change (0–10), readiness to change (0–10)
Session 2	n/a
SM3	Number of binges, belief in change (0–10), readiness to change (0–10)
Session 3	n/a
SM4	Number of binges, belief in change (0–10), readiness to change (0–10)
Session 4	n/a
SM5	Number of binges, belief in change (0–10), readiness to change (0–10)
Session 5	n/a
SM6	Number of binges, belief in change (0–10), readiness to change (0–10)
Session 6	n/a
SM7	Number of binges, belief in change (0–10), readiness to change (0–10)
Session 7	n/a
SM8	Number of binges, belief in change (0–10), readiness to change (0–10)
Session 8	n/a
SM9	Number of binges, belief in change (0–10), readiness to change (0–10)
Session 9	n/a
SM10	Number of binges, belief in change (0–10), readiness to change (0–10)
Session 10	n/a
T2 – Measures at the end of treatment	BED-Q, EDE-Q, MDI

Only measures relevant to the present study are included. For example, patients also reply to the Client Satisfaction Questionnaire at T2, but this is not included in the analyses for this study and is therefore excluded from this overview.

BED-Q, binge eating disorder-questionnaire; EDE-Q, eating disorder examination questionnaire; MDI, major depression inventory; SMx, symptom monitoring x; Tx, time x.

### Eating disorder examination questionnaire

The Eating Disorder Examination Questionnaire (EDE-Q) is a self-report questionnaire assessing the severity and type of eating disorder symptoms and psychopathology during the preceding 28 days (22). The questionnaire comprises 22 items measured on a seven-point Likert scale (0: no days to 6: every day) and six open questions. It produces four subscales: Restraint (mean of items 1–5), Eating Concern (mean of items 7, 9, 19–21), Shape Concern (mean of items 6, 8, 10–11, 23, 26–28), Weight Concern (mean of items 8, 12, 22, 24–25), and a composite scale, the EDE-Q Global scale, defined as the mean of the four subscales. Since all EDE-Q scales are mean scales, they have a range of 0–6. The EDE-Q Global scale can be used to generate eating disorder diagnoses. The current version is designed to generate DSM-5 compatible eating disorder diagnoses (23, 24). Only the EDE-Q Global scale is considered in the current paper.

### Binge eating disorder questionnaire

The Binge Eating Disorder Questionnaire (BED-Q) is a nine-item questionnaire exploring the patients’ BED symptoms (25). The scale measures the presence and severity of symptoms on a global scale from 0 (no BED symptoms) to 35 (symptoms and possible indication of extreme BED). For each of the nine items, the patient is asked to rate how many times per week they experience different symptoms of BED, such as binge eating episodes, loss of control, experiencing eating faster, and so forth. The rate is placed on a Likert scale from 0 (no times per week) to 5 (14 + times per week).

### Major depression inventory

The Major Depression Inventory (MDI) is a 10-item questionnaire pertaining to the ICD-10 and DSM-5 symptoms of depression illness (24, 26). For each of the three final items (items 8–10), the item is divided into two sub-items, of which only the sub-item with the highest score is included. The scale measures items on a six-point scale from 0 (never) to 5 (all the time) over the last 2 weeks. The MDI global score ranges from 0 (no symptoms of depression) to 50 (extreme symptoms of depression) and describes the severity of depressive symptoms present in the patient.

### Statistics

The internal consistencies of the MDI and BED-Q scales and the four EDE-Q subscales were assessed using Cronbach’s α (27). As the EDE-Q global scale is an average of four subscales with different numbers of items and one item occurring in two subscales, Cronbach’s α is inappropriate.

Baseline characteristics of participants who completed the treatment and participants who did not complete treatment were compared using chi-square tests for categorical covariates (sex) and Wilcoxon rank sum tests for continuous covariates (age, BMI, MDI, EDE-Q Global, BED-Q, number of binge eating episodes during the past 7 days, belief in change, and readiness for change).

Associations between treatment completion (defined as completing at least eight out of the ten treatment sessions) and measures of *Belief in change* and *Readiness for change* at Session 1 were analyzed using logistic regression with robust standard errors. The analyses were adjusted for the potential confounders: sex, age, BMI, and MDI at T1.

Analyses concerning the patients’ early treatment measures of *Belief in change* and *Readiness for change* were conducted in two steps. First, individual linear regression slopes for each patient were estimated as increase in belief in change per 10 days during the time interval (in days) from Sessions one to five and increase in *Readiness to change* per 10 days during the same time interval. Second, associations between treatment completion and patient regression slopes were analyzed using logistic regression with robust standard errors, adjusting for sex, age, BMI, and MDI at T1, and for the given measure at Session 1.

Secondary outcomes comprised EDE-Q global score at T2, BED-Q score at T2, and weekly number of binge eating episodes at Session 10. Associations between the secondary outcomes and *Belief in change* and *Readiness for change* at Session 1, as well as patient regression slopes of *Belief in change* and *Readiness to change*, were analyzed using linear regression, adjusting for sex, age, BMI, and MDI at T1. Analyses concerning slopes were further adjusted for the given measure at Session 1. Due to moderate deviations from model assumptions on homogeneity and normality of residuals in the analyses on weekly number of binge eating episodes, robust standard errors were used in analyses on this outcome.

For all analyses, model assumptions were assessed using deviance residual plots for logistic regression analyses and residual plots and normal probability plots for linear regression analyses. The analyses estimating patient regression slopes indicated a pronounced outlier with respect to both belief in change and readiness to change. Sensitivity analyses, excluding the outlier, were conducted for all analyses concerning regression slopes.

All analyses were performed in STATA 17.0 (StataCorp, Texas, USA).

## Results

The internal consistency was good for the MDI and BED-Q scales and the EDE-Q Restraint, Shape concern, and Eating concern subscales (all αs between 0.78 and 0.89), while the EDE-Q Weight concern subscale was only moderate (α = 0.66).

In the total BED sample (*n* = 153), we found high mean scores of treatment motivation (Belief in change = 6.7, Readiness for change = 8.6) at baseline. We found a high mean score of BMI (37.8), a moderate mean score of MDI (23.1), and medium to high mean scores of ED symptomatology (EDE-Q Global = 3.7, BED-Q = 17.2, number of binge eating episodes = 3.8) (see Table 1). None of the baseline characteristics differed statistically significantly between those completing and those not completing, apart from belief in change, which is analyzed in detail below.

Next, we examined the association between treatment motivation and treatment completion. We did not find that baseline measures of treatment motivation predicted treatment completion. However, considering the regression slope from sessions 1 to 5 (early measures), *Belief in Change* did show a statistically significant association with treatment completion. For each one unit increase in the *Belief in change* slope, the odds of completing treatment increased 2.18 times [see Table 2, OR (95%-CI) = 2.18 (1.06, 4.46)]. *Readiness for change* did not show a significant association.

Subsequently, we examined the association between treatment motivation and eating disorder outcomes (EDE-Q Global, BED-Q, and number of weekly binge eating episodes). We did not find that baseline measures of treatment motivation predicted symptom reduction in any eating disorder outcomes. However, the regression slope from sessions 1–5 of *Belief in change* showed a statistically significant association with symptom reduction in regard to EDE-Q Global and weekly number of binge eating episodes (EDE-Q Global: *B* = −0.53, *p* = 0.001; number of weekly binge eating episodes: *B* = 0.81, *p* < 0.01) indicating greater symptom reduction with greater increase in *Belief in change* from Sessions 1–5. BED-Q did not show significant associations (see Table 3). The slope regression from sessions 1–5 for *Readiness to change* showed a statistically significant association with symptom reduction in EDE-Q Global (*B* = −0.46, *p* < 0.05) but not in other eating disorder outcomes.

## Discussion

The study aimed to investigate the associations between treatment motivation, treatment completion, and symptom reduction in completers.

One of the main findings in this study was that baseline treatment motivation did not significantly predict treatment completion. This contradicts findings from existing literature that indicates that low levels of motivation tend to result in higher drop-out rates in treatment of eating disorders (18). For example, Vall and Wade (20) found a mean correlation of 0.23 between treatment motivation and drop-out. However, other studies have shown inconclusive results of baseline treatment motivation as a predictor of treatment adherence in eating disorders (28, 29).

Much of this ambiguity may be explained by the heterogeneity of study designs and measures addressing treatment motivation in eating disorders (20). For example, Aardoom et al. (30) used three items, answered on a 10-point Likert scale at baseline, while Bewell and Carter (15) used only one item, measured 4 weeks into treatment. Other studies have used screening instruments consisting of up to 72 items and other assessment tools such as interviews (18). In our study, treatment motivation was comprised of two questions on a 10-point Likert scale addressing *Readiness to change* and *Belief in change*. This also yielded ambiguous results, as only *Belief in change* had a significant ability to predict treatment completion. This may call into question the construct validity of the items used. To remedy this ambiguity, reaching common scientific ground on measuring treatment motivation is necessary. This could perhaps be achieved by conducting further research on the topic with more reliable and validated measures. However, no validated measures or scales assessing treatment motivation specifically in relation to BED have been put forward in the literature. This may partly be due to the fact that BED research is still in its early stages.

Another factor that might explain the deviating findings of our study could be the heterogeneity of eating disorders examined in previous studies. Here, the majority have emphasized treatment motivation with regard to Anorexia Nervosa and Bulimia Nervosa (29). A scarce amount of studies have focused on treatment motivation in BED (18), which notably differs in its etiology and nature. In a clinical setting, treatment motivation in patients with Anorexia Nervosa can be a major challenge due to their ego-syntonic experience of the disorder, causing them to value their symptoms as part of their personality and belief system (31). As a result, patients show a tendency of low engagement in treatment and denial of problems (32). However, the phenomenon of ego-syntonicity is most prominent in patients suffering from Anorexia Nervosa, while patients suffering from Bulimia Nervosa and BED mainly have an ego-dystonic nature. Opposite to ego-syntonicity, ego-dystonicity is characterized by the capability to differentiate symptoms of the disorder from their self-concept and acknowledge these as a problem (33). Ego-dystonicity may implicitly alleviate the issue of low treatment motivation in some ways and may explain why the baseline treatment motivation was high in our study. From a clinical perspective, this finding brings positive implications, as literature has shown that patients with higher baseline motivation have better chances of achieving positive treatment outcomes. However, the high baseline scores of treatment motivation may also have caused a ceiling effect for the statistical analyses, which might explain the inability to predict treatment completion. Future studies should consider other measures in their research design to overcome this problem, which could possibly be repeatedly evident for this specific population. In either case, what is apparent is that the sample of patients with BED is very motivated for engaging in treatment. This indicates an unmet need; as usual, you would expect a more heterogeneous motivation spread.

The self-referral format may also be a contributing factor to the discrepancy between the literature and the results. It may be that a wider variety of patient motivation would occur in samples that did not self-refer, which could impact the correlation between adherence and motivation. However, this also opens the possibility that the online format can reach a population of highly motivated patients who are not currently undergoing treatment but need it. It begs the question – if they are so highly motivated, why are they not already in treatment? Perhaps this is due to the distinction between motivation for seeking treatment and motivation for engaging in treatment, which could be further investigated in future studies.

Looking beyond baseline scores, early measures of treatment motivation showed a significant ability to predict treatment outcomes. This is in concordance with findings from a previous study, which indicated that early measurements and early symptom change robustly predict post-treatment outcomes in eating disorders (20). However, *Belief in change* seemed also, in this instance, to be the strongest predictor compared to *Readiness to change*. Furthermore, the significance threshold was reached only for the outcomes *EDE-Q* and *Weekly number of binge eating episodes*. This leaves questions about why treatment motivation could not predict symptom reduction in *BED-Q*. Yet again, this indicates that the reader must interpret the results with caution.

From a clinical perspective, the ability of early measures of treatment motivation to predict symptom reduction emphasizes the importance of monitoring patients’ symptoms during treatment to address the potential risk of retention. This finding could perhaps be transdiagnostically transferred to other psychiatric populations. For example, Forsell et al. (34) identified patients at risk of retention halfway through the course of iCBT for insomnia by monitoring their symptoms and randomly assigning them to either continue standard iCBT or to an adapted iCBT group. They found that the latter group was superior in terms of reducing symptoms. This shows how retention could be prevented, and tailoring patients’ needs may enhance treatment effectiveness. More research on treatment motivation in BED is needed to support the findings of this study.

### Limitations

The present study does not have a control group, wherefore comparisons to other groups are purely hypothetical and should be tested further.

The self-referral format does possibly preclude patients with lower motivational scores from participation, which means that the results are primarily generalizable in samples of self-referred patients.

Another limitation to the study was the use of non-standardized between-session measures (*Belief in change* and *Readiness for change*), which originally were included for clinical purposes. To this date, there exists no gold standard for measuring treatment motivation in BED patients.

Finally, self-report measures can be beneficial for reflecting internal and subjective concepts, such as motivation to engage in treatment; however, some literature suggests that treatment engagement itself is more validly measured by therapist ratings (14). Thus, future studies may benefit from utilizing additional types of outcome measures, such as more objective measures, to evaluate treatment motivation.

## Data availability statement

The datasets presented in this article are not readily available due to privacy or ethical restrictions. The data that support the findings of this study are available on request from the corresponding author.

## Ethics statement

Ethical review and approval was not required for the study on human participants in accordance with the local legislation and institutional requirements. The patients/participants provided their written informed consent to participate in this study.

## Author contributions

ER, EJ, ML, KM, and PL contributed to the conception and design of the study. ER, EJ, and SH organized the database. PL performed the statistical analyses. ER wrote the first draft of the manuscript. ER, EJ, ML, KM, PL, and SH wrote the sections of the manuscript. All authors contributed to the manuscript revision, read, and approved the final edition for submission.

## References

[1] Erskine HE Whiteford HA. Epidemiology of binge eating disorder. *Curr Opin Psychiatry.* (2018) 31:462–70. 10.1097/YCO.0000000000000449 30113324

[2] Hudson JI Hiripi E Pope HG Jr. Kessler RC. The prevalence and correlates of eating disorders in the national comorbidity survey replication. *Biol Psychiatry.* (2007) 61:348–58. 10.1016/j.biopsych.2006.03.040 16815322 PMC1892232

[3] Galmiche M Dechelotte P Lambert G Tavolacci MP. Prevalence of eating disorders over the 2000-2018 period: a systematic literature review. *Am J Clin Nutr.* (2019) 109:1402–13. 10.1093/ajcn/nqy342 31051507

[4] American Psychiatric Association. American psychiatric association. *Diagn Statist Manual Mental Dis.* (2013) xxvii:886.

[5] McElroy SL Guerdjikova AI Mori N Munoz MR Keck PE. Overview of the treatment of binge eating disorder. *CNS Spectr.* (2015) 20:546–56. 10.1017/S1092852915000759 26594849

[6] Linardon J Shatte A Messer M Firth J Fuller-Tyszkiewicz M. E-mental health interventions for the treatment and prevention of eating disorders: an updated systematic review and meta-analysis. *J Consult Clin Psychol.* (2020) 88:994–1007. 10.1037/ccp0000575 32852971

[7] Moghimi E Davis C Rotondi M. The efficacy of ehealth interventions for the treatment of adults diagnosed with full or subthreshold binge eating disorder: systematic review and meta-analysis. *J Med Internet Res.* (2021) 23:e17874. 10.2196/17874 34283028 PMC8335602

[8] Barlow DH Bullis JR Comer JS Ametaj AA. Evidence-based psychological treatments: an update and a way forward. *Ann Rev Clin Psychol.* (2013) 9:1–27. 10.1146/annurev-clinpsy-050212-185629 23245338

[9] Cuijpers P. Four decades of outcome research on psychotherapies for adult depression: an overview of a series of meta-analyses. *Can Psychol Psychol Can.* (2017) 58:7–19. 10.1037/cap0000096

[10] Regan P Cachelin FM Minnick AM. Initial treatment seeking from professional health care providers for eating disorders: a review and synthesis of potential barriers to and facilitators of “first contact”. *Int J Eat Disord.* (2017) 50:190–209. 10.1002/eat.22683 28134980

[11] Ali K Farrer L Fassnacht DB Gulliver A Bauer S Griffiths KM. Perceived barriers and facilitators towards help-seeking for eating disorders: a systematic review. *Int J Eat Disord.* (2017) 50:9–21. 10.1002/eat.22598 27526643

[12] Melville KM Casey LM Kavanagh DJ. Dropout from internet-based treatment for psychological disorders. *Br J Clin Psychol.* (2010) 49:455–71. 10.1348/014466509X472138 19799804

[13] Dölemeyer R Tietjen A Kersting A Wagner B. Internet-based interventions for eating disorders in adults: a systematic review. *BMC Psychiatry.* (2013) 13:207. 10.1186/1471-244x-13-207 23919625 PMC3750530

[14] Drieschner KH Lammers SM van der Staak CP. Treatment motivation: an attempt for clarification of an ambiguous concept. *Clin Psychol Rev.* (2004) 23:1115–37. 10.1016/j.cpr.2003.09.003 14729425

[15] Bewell CV Carter JC. Readiness to change mediates the impact of eating disorder symptomatology on treatment outcome in anorexia nervosa. *Int J Eat Disord.* (2008) 41:368–71. 10.1002/eat.20513 18306345

[16] Clausen L Lubeck M Jones A. Motivation to change in the eating disorders: a systematic review. *Int J Eat Disord.* (2013) 46:755–63. 10.1002/eat.22156 23847134

[17] Denison-Day J Appleton KM Newell C Muir S. Improving motivation to change amongst individuals with eating disorders: a systematic review. *Int J Eat Disord.* (2018) 51:1033–50. 10.1002/eat.22945 30189116

[18] Hoetzel K von Brachel R Schlossmacher L Vocks S. Assessing motivation to change in eating disorders: a systematic review. *J Eat Disord.* (2013) 1:38. 10.1186/2050-2974-1-38 24999416 PMC4081820

[19] Linardon J Hindle A Brennan L. Dropout from cognitive-behavioral therapy for eating disorders: a meta-analysis of randomized, controlled trials. *Int J Eat Disord.* (2018) 51:381–91. 10.1002/eat.22850 29493805

[20] Vall E Wade TD. Predictors of treatment outcome in individuals with eating disorders: a systematic review and meta-analysis. *Int J Eat Disord.* (2015) 48:946–71. 10.1002/eat.22411 26171853

[21] Webb CA Rosso IM Rauch SL. Internet-based cognitive-behavioral therapy for depression: current progress and future directions. *Harv Rev Psychiatry.* (2017) 25:114–22. 10.1097/HRP.0000000000000139 28475503 PMC5421393

[22] Fairburn CG Beglin SJ. Assessment of eating disorders: interview or self-report questionnaire? *Int J Eat Disord.* (1994) 16:363–70. 7866415

[23] Fairburn CG. *Cognitive Behavior Therapy and Eating Disorders.* New York: Guilford Press (2008).

[24] World Health Organization. *The ICD-10 Classification of Mental and Behavioural Disorders. Clinical Descriptions And Diagnostic Guidelines.* Geneva: World Health Organization (1992).

[25] Jensen ES Linnet J Holmberg TT Tarp K Nielsen JH Lichtenstein MB. Effectiveness of internet-based guided self-help for binge-eating disorder and characteristics of completers versus noncompleters. *Int J Eat Disord.* (2020) 53:2026–31. 10.1002/eat.23384 32918321

[26] Bech P. *Clinical Psychometrics.* West Sussex: John Wiley & Sons, Ltd. (2012).

[27] Bland JM Altman DG. Cronbach’s alpha. *BMJ.* (1997) 314:572. 10.1136/bmj.314.7080.572 9055718 PMC2126061

[28] Abd Elbaky GB Hay PJ le Grange D Lacey H Crosby RD Touyz S. Pre-treatment predictors of attrition in a randomised controlled trial of psychological therapy for severe and enduring anorexia nervosa. *BMC Psychiatry.* (2014) 14:69. 10.1186/1471-244x-14-69 24606873 PMC3995934

[29] Sansfacon J Booij L Gauvin L Fletcher E Islam F Israel M Pretreatment motivation and therapy outcomes in eating disorders: a systematic review and meta-analysis. *Int J Eat Disord.* (2020) 53:1879–900. 10.1002/eat.23376 32954512

[30] Aardoom JJ Dingemans AE Spinhoven P Hakkaart-van Roijen L Van Furth EF. An internet-based intervention for eating disorders consisting of automated computer-tailored feedback with or without supplemented frequent or infrequent support from a coach: study protocol for a randomized controlled trial. *Trials.* (2013) 14:340. 10.1186/1745-6215-14-340 24135131 PMC3853403

[31] Dray J Wade TD. Is the transtheoretical model and motivational interviewing approach applicable to the treatment of eating disorders? *Rev Clin Psychol Rev.* (2012) 32:558–65. 10.1016/j.cpr.2012.06.005 22819997

[32] Gregertsen EC Mandy W Serpell L. The egosyntonic nature of anorexia: an impediment to recovery in anorexia nervosa treatment. *Front Psychol.* (2017) 8:2273. 10.3389/fpsyg.2017.02273 29312100 PMC5743910

[33] Fairburn CG. *Overcoming Binge Eating. The Proven Program to Learn Why You Binge and How You Can Stop.* 2nd ed. New York & London: The Guilford Press (2013).

[34] Forsell E Jernelöv S Blom K Kraepelien M Svanborg C Andersson G Proof of concept for an adaptive treatment strategy to prevent failures in internet-delivered CBT: a single-blind randomized clinical trial with insomnia patients. *Am J Psychiatry.* (2019) 176:315–23. 10.1176/appi.ajp.2018.18060699 30696270

